# Polypropylene Glycol-Polyoxytetramethylene Glycol Multiblock Copolymers with High Molecular Weight: Synthesis, Characterization, and Silanization

**DOI:** 10.3390/molecules24234317

**Published:** 2019-11-26

**Authors:** Wei Hu, Lei Wang, Quanyong Wang, Anbo Luan, Yuliang Mai, Linjia Huang, Yongjun Chen, Shijing Yan, Wenjie Xiong

**Affiliations:** 1 Guangdong Research Institute of Petrochemical and Fine Chemical Engineering, Guangzhou 510665, China; gracehz@126.com (W.H.);; 2 Guangdong Province Key Laboratory of High Performance and Functional Polymer materials, South China University of Technology, Guangzhou 510640, China; 3 School of Chemistry and Chemical Engineering, Guangxi University for Nationalities, Nanning 530006, China

**Keywords:** polypropylene glycol-polyoxytetramethylene glycol block multicopolymers, chain extender, m-phthaloyl chloride, adhesives, structure optimizing

## Abstract

The high crystallization at room temperature and high cost of polyoxytetramethylene glycol (PTMG) have become obstacles to its application. To overcome these problems, a segment of PTMG can be incorporated into a block copolymer. In this work, polypropylene (PPO) glycol-polyoxytetramethylene (PPO-PTMG) multiblock copolymers were designed and synthesized through a chain extension between hydroxyl (OH)-terminated PPO and PTMG oligomers. The chain extenders, feed ratios of the catalyst/chain extender/OH groups, reaction temperature, and time were optimized several times to obtain a PPO-PTMG with low crystallization and high molecular weight. Multiblock copolymers with low crystallization and high average molecular weight (*M_n_* = 1.0–1.4 × 10^4^ Dalton) were harvested using m-phthaloyl chloride as the chain extender. The OH-terminated PPO-PTMG multiblock copolymer with high *M_n_* and a functionality near two was further siliconized by 3-isocyanatopropyltrimethoxysilane to synthesize a novel silyl-terminated polyether. This polyether has an appropriate vulcanizing property and potential applications in sealants/adhesive fields.

## 1. Introduction

Materials based on polyoxytetramethylene glycol (PTMG) such as polyurethane (PU) and thermoplastic poly(ether ester) elastomers have superior mechanical properties, oil resistance, abrasion resistance, hydrolytic stability, and anti-aging properties than those based on polyethylene glycol (PEO) and polypropylene (PPO) [1,2]. However, the high crystallization at room temperature and high cost of PTMG have become obstacles to its application. Tetrahydrofurane-epoxide copolymers have been developed to replace PTMG since the 1960s to improve the production, processability, and low-temperature performance and reduce the cost. The tetrahydrofurane-epoxide copolymer is a type of block polyether and is mostly synthesized by the cationic ring opening polymerization of tetrahydrofurane and epoxides including ethylene oxide (EO) [3], propylene epoxide (PO) [4], epichlorohydrin (ECH) [5,6,7], and cyano EO [8,9,10]. Generally, these copolymers, especially PPO-PTMG multiblock copolymers, possess a medium molecular weight (2000–4000 Dalton) [11,12]. This condition results from the fact that the further chain propagation of copolymers is inhibited by the low ring opening reactivity of tetrahydrofurane [13,14,15] and the chain transfer of cationic ring opening polymerization [16]. Hence, these methods show no difference from the traditional approach of chain extension in obtaining a high molecular weight (*M_n_* > 10^4^ Dalton) of PU or poly(ether ester) products [17,18].

Increasing the molecular weight of block polyether based on PTMG to broaden its application has become an issue. Wang et al. [19] and Zhang et al. [20] proposed a new strategy of using PTMG oligomers as a macroinitiator to trigger the ring opening polymerization of PO and EO, respectively. This strategy avoids using low reactive tetrahydrofurane, and the highest *M_n_* of the resulting corresponding block polyethers could reach 4817 Dalton and 7370 Dalton. By adopting PTMG oxonium ions to couple hydroxyl (OH)-terminated PTMG (*M_n_* = 1000 Dalton) and PPO (*M_n_* = 650 Dalton) oligomers, a PPO-PTMG block copolymer was also synthesized and had a considerable *M_n_* (8156 Dalton) [21]. The two methods are helpful in increasing the *M_n_* of the block polyether compared with the former cationic ring opening polymerization of epoxy monomers. Nevertheless, they are still not aligned with the expectations of *M_n_* higher than 10^4^ Dalton for the relative low reactivity of PTMG initiators or oxonium ions. On the basis of the two methods and the synthesis methods of PUs or poly(ether ester)s, the chain extension of PTMG and other polyether polyol oligomers using high reactive chain extenders could gain a high molecular weight PTMG-based block polyether [22]. The resulting block polyether with terminal OH groups would be further functionalized to generate special properties and new applications.

In this work, we focused on the synthesis of PPO-PTMG multiblock copolymers with a high molecular weight through chain extension, and focused on the modification of the OH-terminated block copolymer by silanization. Highly reactive chain extenders (e.g., saturated dihalide and aliphatic and aromatic dicarbonyl chlorides (except diisocyanate due to its high toxicity)) were selected for chain extension between OH-terminated PTMG and PPO oligomers. The chain extension effects were evaluated through several analysis methods. Next, the influences of the feed ratios of the deacid reagent/chain extender/OH groups, the reaction temperature, and time on the molecular weight of the block copolymers were also investigated by gel permeation chromatography (GPC). Finally, one OH-terminated product with a high *M_n_* was selected for terminal silicone modification, and the modified product used as the novel base polymer in sealants/adhesive was preliminarily appraised by its vulcanizing properties.

## 2. Results and Discussion

### 2.1. Choice of Chain Extenders

Three chain extenders, namely, 1,2-dichloromethane, sebacoyl chloride, and m-phthaloyl chloride were used in the coupling of the PPO and PTMG oligomers. The corresponding products, denoted as copolymers-I, -II, and -III, were characterized by the Nuclear magnetic resonance (^1^H-NMR) and the Fourier transform infrared (FTIR).

**Figure 1:**
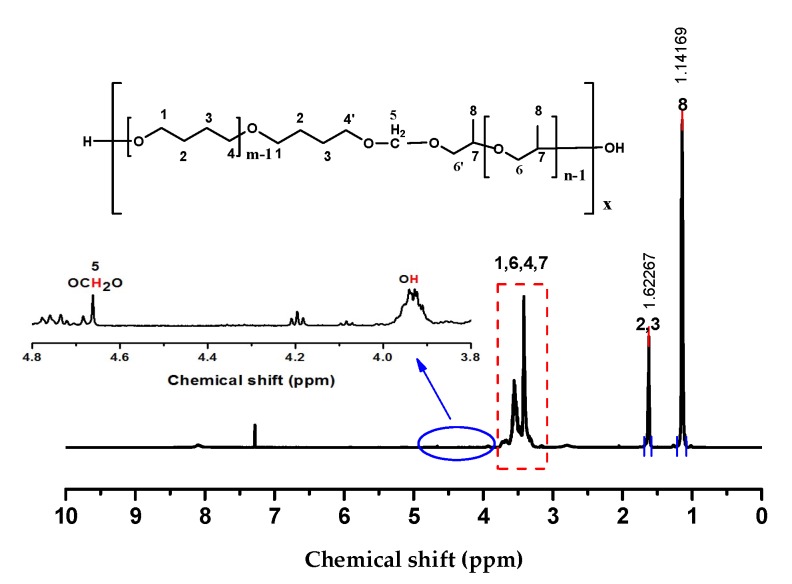
^1^H-NMR spectra of copolymer-I and the assignment of the protons.

**Figure 2:**
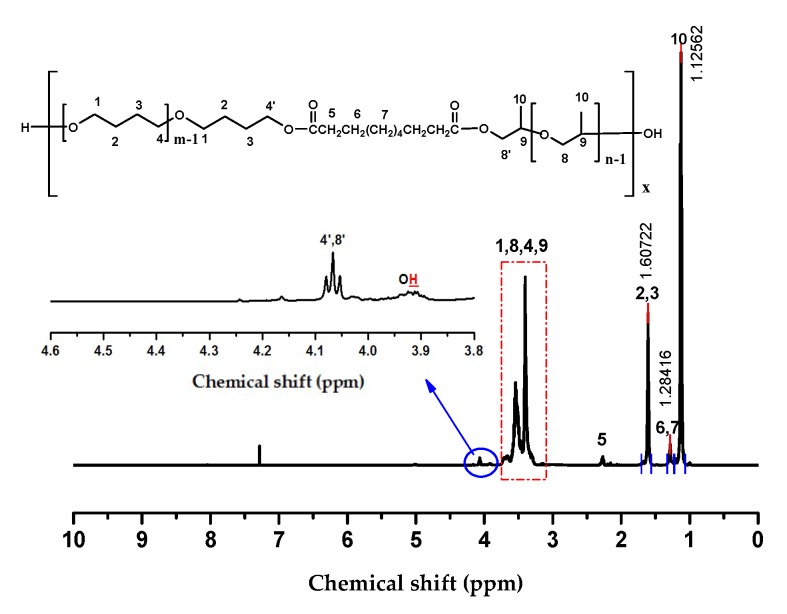
^1^H-NMR spectra of copolymer-II and the assignment of the protons.

**Figure 3:**
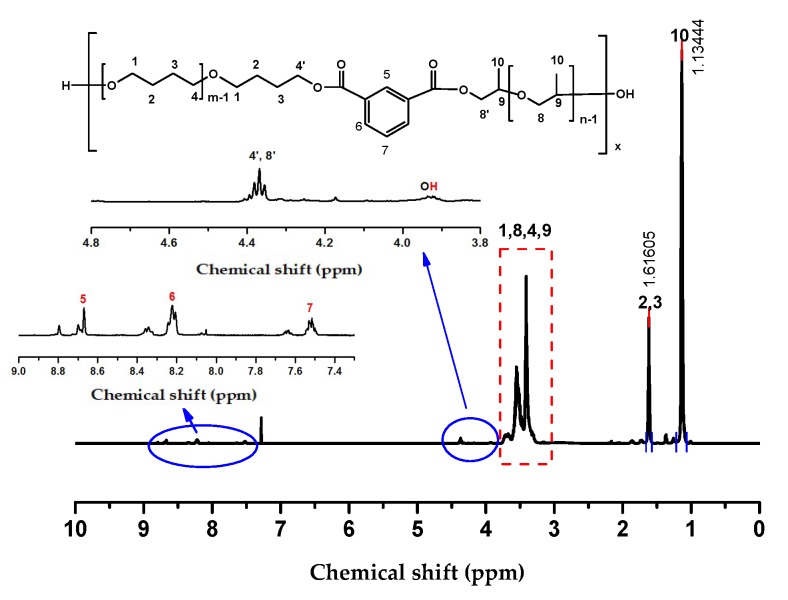
^1^H-NMR spectra of copolymer-III and the assignment of the protons.

Figure 1, Figure 2 and Figure 3 present the ^1^H-NMR spectra of all copolymers and the assignments of proton type. The resonance signals of protons from the methylene (6-H), the methyne (7-H) in the main chain of PPO, and the methylene (1,4-H) bonded to the oxygen atom in the main chain of PTMG overlap in the range of 3.0–3.8 ppm. The protons from the methyl (8-H) and another methylene (2,3-H) in the constitutional units of PPO and PTMG were also observed at 1.14 ppm and 1.62 ppm, respectively. In addition to these characteristic proton peaks from the PPO and PTMG segments, Figure 1, Figure 2 and Figure 3 also indicate the coupling structures in the block copolymers. The inset in Figure 1 shows that the coupling structure of OCH_2_O (5-H) was confirmed by the peak at 4.66 ppm. Likewise, the inset in Figure 2 identifies the coupling structures in copolymer-II by the peaks at approximately 1.21 ppm (6-H and 7-H of copolymer-II), 2.27 ppm (5-H of copolymer-II), and 4.07 ppm (4′-H and 8′-H of copolymer-II). The inset in Figure 3 shows the coupling structures of meta phenyl in copolymer-III, corresponding to the peaks in the range of 7.3–8.8 ppm (Ar–H of copolymer-III). In addition, the methylene (4′-H and 8′-H) from the PPO and PTMG covalent to the ester group is recognized by the peaks at 4.07 ppm and 4.35 ppm for copolymers-II and -III, respectively. Notably, all the block copolymers have a small resonance peak at approximately 3.92 ppm, which belongs to the terminal OH groups. 

As the resonance signals of the protons of the methyl from the PPO segment and the dimethylene (-CH_2_CH_2_-) from the PTMG segment shown in ^1^H-NMR spectra are independent, the relative content of the repeat unit of the PPO and the PTMG can be calculated based on the area (A) of their proton peaks. Figure 1 presents the areas of the methyl (8-H, A8-H) and the dimethylene (-CH_2_CH_2_-, 2,3-H, A2,3-H) peaks from copolymer-I, which were 1.14 and 1.62, respectively. The ratio of A8-H/A2,3-H was 1.42, which could further suggest that the mole content of the repeat unit of the PTMG was more than that of the PPO. Similarly, the calculated ratios of A10-H/A2,3-H from copolymers-II and -III were 1.27 and 1.43, respectively. The mole content of the repeat unit of the PTMG was less than that of the PPO for copolymer-II, and the opposite for copolymer-III.

**Figure 4:**
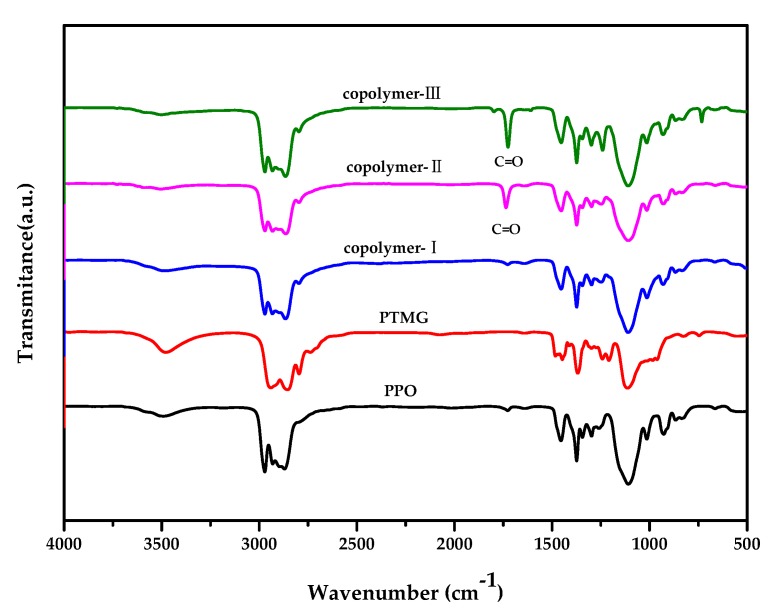
FTIR spectra of the copolymers and two raw materials.

The structures of the block copolymers were further characterized by the FTIR spectra, and the results are shown in Figure 4. The intensity of the absorption bands in the range of 3250–3750 cm^−1^ for all the block copolymers was considerably weaker than that for PPO and PTMG. Thus, the terminal OH groups were partly consumed in the coupling reactions. Except for the range of 3250–3750 cm^−1^, the FTIR spectra of the three copolymers appeared to be the spectrum superpositions of PPO and PTMG and show additional characteristic absorption peaks at approximately 1730 cm^−1^. These characteristics can be attributed to the C=O bond in the coupling structure for copolymers-II and -III. These results are consistent with the structure composition of copolymers.

**Figure 5:**
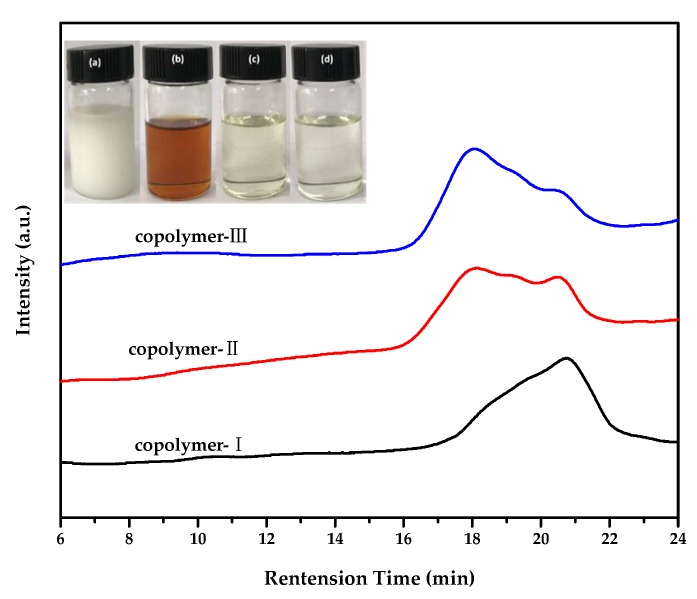
GPC curves for block copolymers. The inset in the top left corner show (a) blends of PPO and PTMG, (b) copolymer-I, (c) copolymer-II, and (d) copolymer-III.

The effects of the three chain extenders were investigated by GPC. As shown in Figure 5, the GPC curve for each copolymer showed multiple peaks, indicating several species with different molecular weights as other copolymers [23]. The calculated values of the average molecular weight (*M_n_*) and dispersity index (Đ = *M_w_*/*M_n_*) of the copolymers that are listed in Table 1 show that copolymers-II and -III possess higher molecular weight and wider molecular weight distribution than copolymer-I. The high reactivity of acid chloride chain extenders can be operated through a simple one-step method under mild conditions, thus avoiding the side reactions of chain scission and oxidation. Therefore, a light-colored high molecular weight (*M_n_* > 10^4^ Dalton) block copolyether (seen in the inset as (c) and (d) in Figure 5) is easily gained.

Table 1 also shows the OHV and *f*. For all copolymers, each titrimetric OHV and *f* was less than their separate theoretical values. These results demonstrate that both ends of the copolymer chains are not ideally terminated by the OH group. In the synthesis processes of copolymers-I, -II, and -III, the side reactions of chain scission and oxidation might be caused by high temperature (120 °C), strong alkali (CH_3_ONa), or the backbiting side reaction from the high reactive end groups (COCl) [20]. The first side reaction is the most evident in the synthesis of copolymer-I due to the strict reaction conditions, which is fully embodied by the deepest color (shown in the inset as (b) in Figure 5), the lowest *M_n_*, the largest OHV deviation, and the lowest *f* value of copolymer-I.

**Figure 6:**
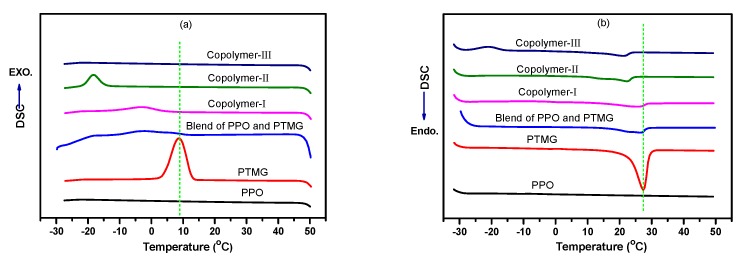
Differential scanning calorimetry (DSC) curves of copolymers at 5 °C/min cooling and heating rate, (**a**) crystallization curves; (**b**) melting curves.

Generally, PPO and PTMG (*M_n_* ≥ 2000 Dalton) are amorphous and crystalline polymers, and their blends present a white crystallizing solid (shown in the inset as (a) in Figure 5) at ambient temperature for PTMG crystallization. In contrast, all block copolymers are in a liquid state at ambient temperature, which indicates that a difference in crystallization property exists between the block copolymers and PTMG. The DSC curves are provided in Figure 6 to illustrate the crystallization property of raw materials and copolymers. PPO shows no enthalpy change, whereas the DSC curve of PTMG shows remarkable crystallization and fusion peaks in the cooling and heating processes, which are separately ascribed to the amorphous and crystalline structures of PTMG, respectively. The crystallization of copolymers is related to the *M_n_* of copolymers. However, the DSC curves of copolymers-I and -II with low *M_n_* showed small crystallization peaks, and their maximal crystallization rate temperatures (*T_c_*, Table 1) were obviously lower than those of PTMG and the PPO and PTMG blend, and decreased with the increase of *M_n_*. Interestingly, the DSC curve of copolymer-III with the highest *M_n_* showed no crystallization peak in its cooling process, but presented a cold crystallization peak concentrated at −20.9 °C in its heating process. The cold crystallization peak is commonly seen in polymers with a low crystallization rate [24], indicating that copolymer-III had the weakest crystallization capability. The broad and small fusion peaks in the heating DSC curves of the block copolymers (Figure 6b) imply that an imperfect crystal structure exists in copolymers, and the imperfect extent increases with *M_n_*, as shown by the order of melting temperature (*T_m_*) of copolymers (Table 1). It is noteworthy that there was a slight difference between the DSC curves of copolymer-I and blend of PPO and PTMG. One reason for that is the low *M_n_* of copolymer-I and another may be the residual unreacted PTMG containing the low activity of CH_2_Cl_2_. The analysis of DSC indicates that the higher the *M_n_* of the block copolymers, the lower their crystallization capability will be. Furthermore, their low crystallization capability can endow copolyethers with a liquid state, low viscosity, good process, and low temperature performance [25], which are all desirable for materials prepared based on polyether polyols. Therefore, the goal to synthesize block copolyether with high *M_n_* and low crystallization capability is most feasibly realized by using m-phthaloyl chloride as the chain extender. 

### 2.2. Optimizing Reaction Conditions

**Figure 7:**
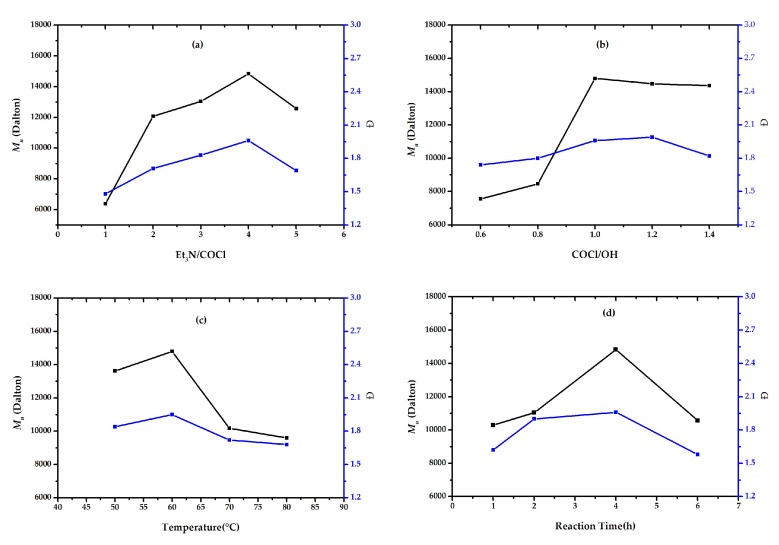
Relationships between *M_n_*, Đ, and feed ratios of (**a**) Et_3_N/COCl, (**b**) COCl/OH, and reaction (**c**) temperature and (**d**) time of copolymer-III.

In Section 2.1, we confirmed that the best chain extending effect was obtained using m-phthaloyl chloride. We further investigated the various factors influencing the *M_n_* and Đ of block copolymers by individual factor experiments to optimize the reaction conditions. First, the influence of the feed ratio of the acid binding agent (triethylamine)/acyl chloride (Et_3_N/COCl) groups was studied. The result is shown in Figure 7a. The *M_n_* and Đ of copolymers increased with the Et_3_N/COCl until the Et_3_N/COCl was equal to 4/1, and the highest *M_n_* and Đ were 14,800 Dalton and 1.96, respectively. Then, *M_n_* and Đ decreased with the further increase of Et_3_N/COCl. This result may be due to the strong alkalinity of the excess Et_3_N that causes the hydrolysis of ester groups in the copolymer chains; the transesterification reaction; or the ester fracture caused by the alkaline environment provided by excessive acid binding agent. The best chain extender dosage was identified after the Et_3_N/COCl feed ratio of 4/1 was determined. Figure 7b shows the relationship between the mole ratio of the COCl/OH groups, and *M_n_* suggests that 1/1 is the appropriate ratio of COCl/OH because the highest *M_n_* (14,800 Dalton) is achieved under this condition. The continuous increase of COCl/OH (>1/1) had no beneficial effect on the increase of the molecular mass. Đ basically kept pace with the change in *M_n_* and decreased slightly when COCl/OH was 1/1.4.

In addition to the dosages of the acid binding agent and the chain extender, the influences of reaction temperature and time on *M_n_* and Đ were also studied. Figure 7c shows that, initially, raising the temperature was conducive to chain extension, and the *M_n_* of copolymers increased from 13,600 Dalton to 14,800 Dalton when the temperature increased from 50 °C to 60 °C, and obviously decreased with the continuous increase of the reaction temperature. Đ had a similar variation rule at the temperature range of 50 °C–80 °C (Figure 7c). One of the reasons for these outcomes can be the acceleration of the hydrolysis of ester groups by the increasing temperature. Another reason might be the exothermic reaction between the COCl groups and activated hydrogen, which is inhibited at high temperature [26]. The other two reasons may be the transesterification reaction and the ester fracture caused by the alkaline environment provided by excessive acid binding agent.

The reaction time was also optimized to obtain the highest *M_n_* and the results are shown in Figure 7d. First, the *M_n_* of the copolymer increased with the extension of the reaction time, and the maximum *M_n_* was observed when the reaction time was 4 h. Then, *M_n_* decreased after 4 h. Figure 7d also exhibits the relationship between Đ and the reaction time. Đ showed a similar variation as *M_n_* and reached the maximum value of less than 2.0 at 4 h. Chain extension was random and heterogeneous; thus, it led to a relatively high Đ of the copolymers. Extending the reaction time from 1 h to 4 h can effectively increase the extent of the coupling reaction and lead to the continuous increase of *M_n_*. However, when extending the time beyond 4 h, chain scission from the hydrolysis of ester groups, thermal oxidation of the terminated OH groups, transesterification reaction, and ester fracture caused by the alkaline environment from the excessive acid binding agent may occur in the synthesis process, which may decrease *M_n_* and deepen the color of the copolymer (not shown here). 

Table 2 lists the *M_n_*, Đ, OHV, and *f* of some block copolyethers. All determined OHV were less than the calculated values, and the difference increased with *M_n_*. Thus, the corresponding *f* calculated from the determined OHV was less than two, and decreased with the increase in *M_n_*. These results suggest that the blocking of the ends of copolymers with the carboxyl groups from the hydrolysis of the COCl groups, transesterification reaction, or ester fracture (caused by the alkaline environment from the excessive acid binding agent) might occur in the synthesis process. Moreover, the blocking effects can be enhanced by the low reactivity of the COCl groups in copolymers with high molecular weight. 

Overall, for the chain extending system using m-phthaloyl chloride as a chain extender, the optimal reaction conditions to achieve high *M_n_* are as follows: Et_3_N/COCl/OH feed ratio of 4/1/1 and reaction temperature and time of 60 °C and 4 h, respectively.

### 2.3. Silanization of Block Polyether

The silyl-terminated polyether resin with high *M_n_* and silane ending ratio are beneficial for preparing high performance silicone modified polyether sealants/adhesives. Thus, the block copolyether with high *M_n_* and a *f* value near two was considered as a good precursor of silyl-terminated polyether resin. In comparison with copolymer-IIIa, copolymers-III, -IIIc, and -IIId are more suitable for silanization to synthesize silicone-modified polyether resins because of their high *M_n_* and *f* values near two (Table 2). In this work, copolymer-III was selected as a precursor and further silanized by 3-isocyanatopropyltrimethoxysilane. The polymer (SiSTP) produced was characterized by ^1^H-NMR and FTIR spectra. 

**Figure 8:**
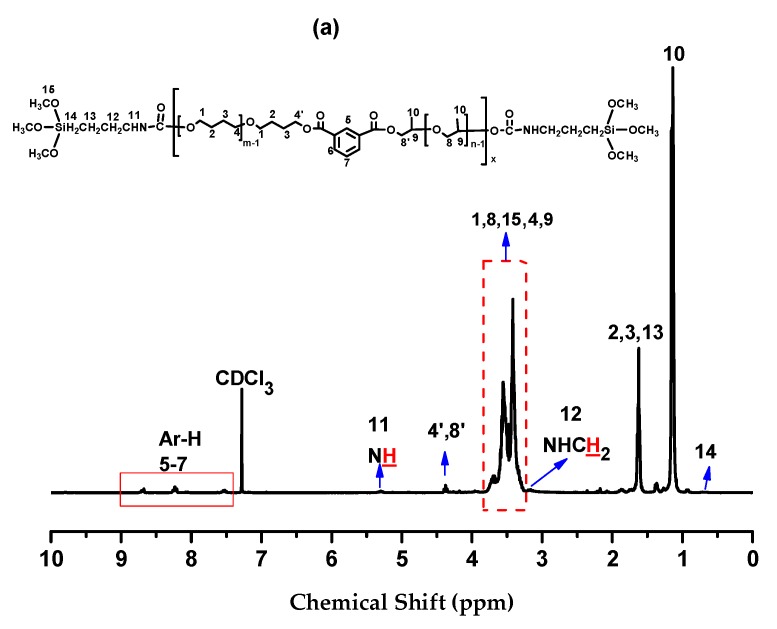

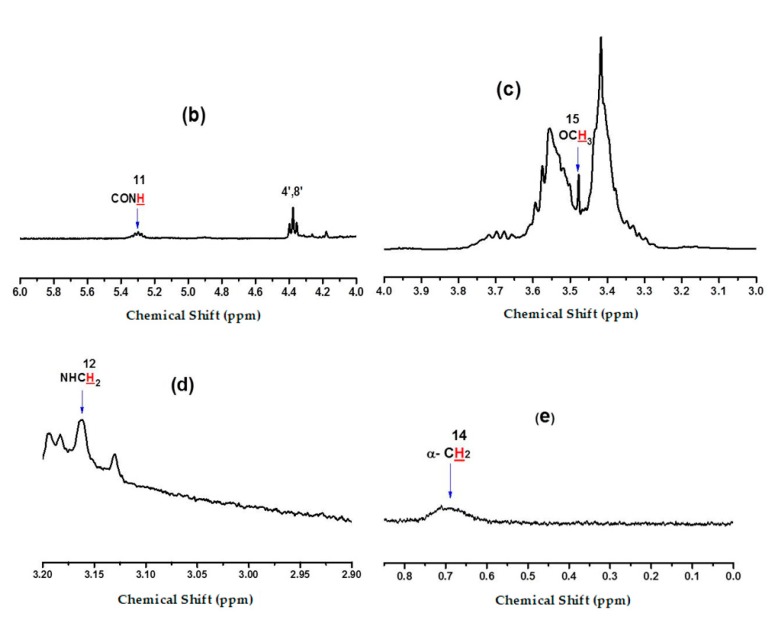
^1^H-NMR spectra of SiSTP: (**a**) full-scan ^1^H-NMR spectrum and close-up of downfield, midfield, and backfield regions of (**a**): (**b**) 4.00–6.00 ppm, (**c**) 3.00–4.00 ppm, (**d**) 2.80–3.20 ppm, and (**e**) 0.00–0.85 ppm.

Figure 8a shows the full-scan ^1^H-NMR spectra of SiSTP. The comparison with the ^1^H-NMR spectra (Figure 3) of copolymer-III showed that the protons (1–10) from the main chain, the methyl side group, and the coupling structure of silyl-terminated copolymer were easily distinguished. The terminated alkoxysilane groups ((CH_3_O)_3_SiCH_2_CH_2_CH_2_) and carbamate (NHCOO) were also confirmed by ^1^H-NMR spectra. The proton peak of methoxyl (15-H) from the alkoxysilane groups was at approximately 3.18 ppm and partially overlapped the proton peaks of the copolymer main chain (Figure 8c). Small bumps at approximately 5.30 ppm and 0.70 ppm were clearly observed in the partial enlargement (Figure 8b,e) and belong to protons (11, 14) from the carbamate and α-methylene in the alkoxysilane groups, respectively. The β-methylene (12-H) in the alkoxysilane groups is displayed as multiple peaks in the range of 3.10–3.20 ppm (Figure 8d). Given that the γ-methylene (13-H) from the alkoxysilane groups and the β-methylene from the PTMG segment have a similar chemical environment, their chemical shifts were extremely close to distinguish and located at approximately 1.62 ppm.

**Figure 9:**
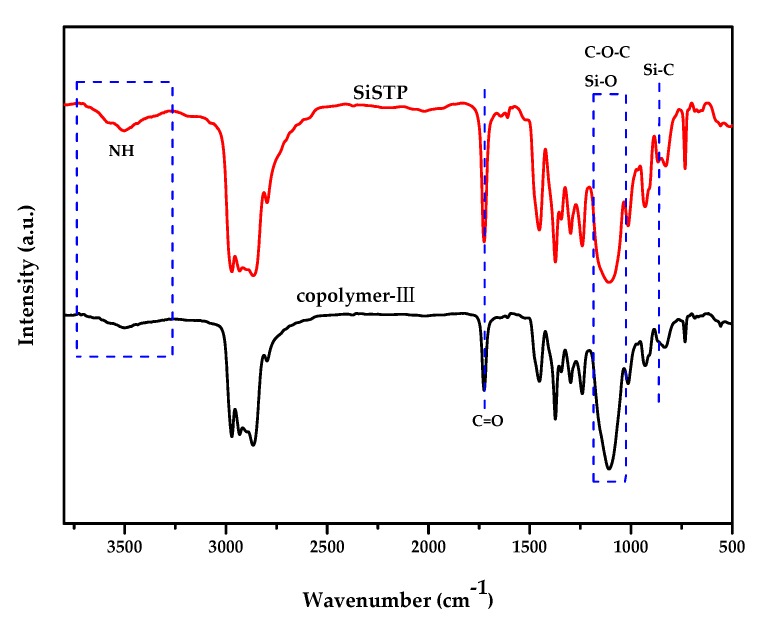
FTIR spectra of SiSTP and copolymer-III.

The FTIR spectra (Figure 9) provides additional evidence for the successful synthesis of silyl-terminated copolyether resin. In comparison with copolymer-III, SiSTP shows absorption bands of the carbonyl group and secondary amine with a stronger intensity at approximately 3500 cm^−1^ and 1725 cm^−1^, respectively, signifying the formation of carbamate. The existence of the Si–C bond is verified by the appearance of a new absorption band concentrated at 866 cm^−1^ in the FTIR spectrum of SiSTP. Moreover, the stretching vibration absorption of the Si–O bond was reflected in the broadening absorption peak at approximately 1108 cm^−1^.

**Figure 10:**
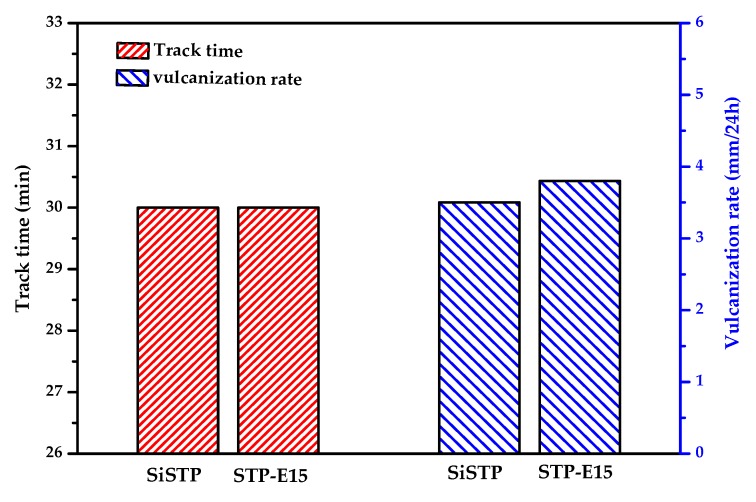
Vulcanization properties of SiSTP and STP-E15.

The vulcanization properties of SiSTP and a commercial silyl-terminated PPO resin, STP-E15, were measured and compared to evaluate the potential application of SiSTP in sealants/adhesives. Figure 10 shows that the track free time of SiSTP was 30 min and equaled that of STP-E15, and the vulcanization rate of SiSTP was up to 3.5 mm/24 h, which was nearly identical to that of STP-E15. STP-E15 has been commonly used as a base polymer in sealants/adhesives. Thus, SiSTP, with similar moist-curable and vulcanization properties, can be an alternative and promising base polymer in sealants/adhesives and improve the properties of sealants/adhesives with its novel and special chemical structure.

## 3. Experimental Section

### 3.1. Materials

PPO (*M_n_* ≈ 4000 Dalton, OH value (OHV) = 28.03 mgKOH/g), PTMG (*M_n_* ≈ 2000 Dalton, OHV = 46.5 mgKOH/g), bentonite, and diatomaceous earth were purchased from Aladdin Holdings Group and used as provided. Sebacoyl chloride, m-phthaloyl chloride, triethylamine (Et_3_N), N-[3-(trimethoxysilyl)propyl]-ethylenediamine, (3-aminopropyl)trimethoxysilane, bis(lauroyloxy)dioctyltin, and ditin butyl dilaurate were provided by Macklin Biochemical Co. Ltd. (Shanghai, China). 3-Isocyanatopropyltrimethoxysilane, bismuth(III) neodecanoate, and anhydrous sodium methoxide were provided by J&K Scientific Ltd. (Guangzhou, China). Dichloromethane and methylbenzene were purchased from Guangzhou Chemical Regent Factory (Guangzhou, China) and used as received. STP-E15 (*M_n_* ≈ 8000–12,000 Dalton), a type of commercial silyl-terminated polyether resin containing PPO glycol and reactive end groups of trimethoxysilylpropylcarbamate, was provided by Wacker Chemie AG (Shanghai, China). 

### 3.2. Synthesis of PPO-PTMG Multiblock Copolyethers Using 1,2-dichloromethane

**Scheme 1:**
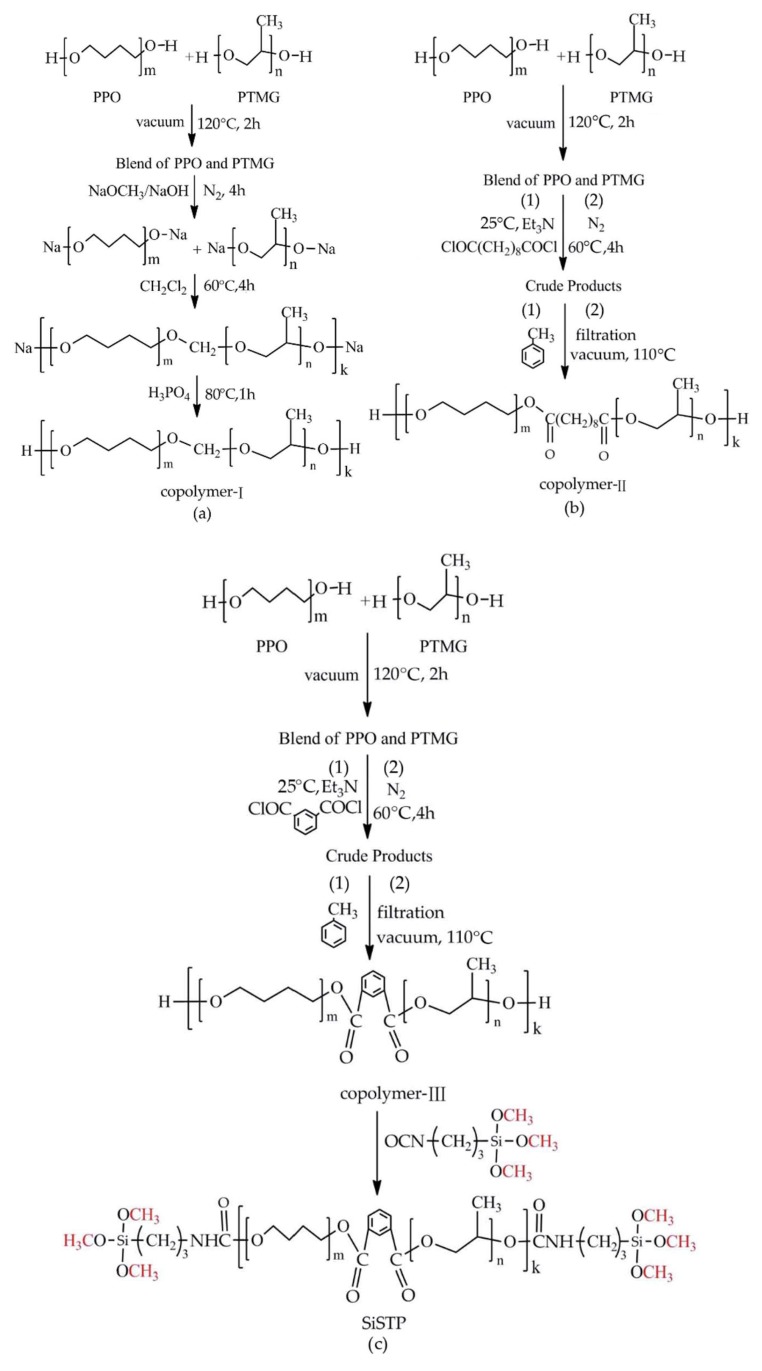
Synthesis of (**a**) copolymer-I, (**b**) copolymer-II, (**c**) copolymer-III and its silanization.

PPG-PTHG multiblocks were synthesized via chain extension, as shown in Scheme 1. When the chain extender was 1,2-dichloromethane, the typical synthesis procedure of PPG-PTHG included two steps of alkoxylation and nucleophilic substitution, described as follows. In a 250-mL three-neck flask, 48.0 g (containing 0.024 mol OH group) of PPO and 12.0 g (containing 0.010 mol OH group) of PTMG were mixed and dried under vacuum at 120 °C for 2 h. Then, 2.623 g (0.049 mol) of alkoxylation reagent of anhydrous sodium methoxide was added to the mixture under vigorous stirring for another 4 h in nitrogen atmosphere to form reactive sodium alkoxide terminal groups in oligomers. The mixture was cooled down to 60 °C, and 3.0534 g (0.036 mol) of dichloromethane was added into the flask. The chain extending reaction was implemented by constantly stirring the mixture under reflux at 60 °C for 4 h. Subsequently, 4.93 g of phosphoric acid was mixed with the crude product and stirred at 80 °C for 1 h to recover the OH groups, and the inorganic salt in crude product was dislodged by the absorption of bentonite (1.84 g) and diatomite (1.84 g) for 6 h. Finally, the mixtures were filtered to obtain the orange-red block copolymer in a viscous liquid state, and this block copolymer was named copolymer-I. The yield of this copolymer was 90%. 

### 3.3. Synthesis of PPO-PTMG Multiblock Copolyethers Using Diacid Chlorides

The synthesis of the PPO-PTMG multiblock copolymer using diacid chlorides as chain extenders is shown in Scheme 1. PPO and PTMG, with the same dosage given in Section 2.2, were mixed and dried under vacuum at 120 °C for 2 h. Then, 4.304 g (0.018 mol) of sebacoyl chloride and 7.286 g (0.072 mol) of Et_3_N were added into the mixture at room temperature. The mixture was heated to 60 °C, and the chain extending reaction was achieved by stirring in a nitrogen atmosphere for 4 h. The crude products were dissolved and diluted with methylbenzene, and the undissolved Et_3_N hydrochloride formed in the reaction was completely removed by filtration. The filtrate was vacuum-rotary evaporated and vacuum-dried at 110 °C for one night to remove methylbenzene thoroughly. Subsequently, a light yellow viscous block copolymer was obtained and recorded as copolymer-II. The yield of this copolymer was 93%

Another multiblock copolymer named copolymer-III containing a benzene coupling structure was obtained by replacing sebacoyl chloride with equimolar m-phthaloyl chloride in the above synthesis procedure. In addition, the influences of the feed ratios of the deacid reagent/chain extender and chain extender/OH groups, the reaction temperature, and time on the molecular weight of block copolymers were studied. The ranges of the factors are listed in Table 3. The corresponding copolymers are collectively referred to as the copolymer-III series. The yields of the copolymers synthesized using m-phthaloyl chloride exceeded 93%. 

### 3.4. Silanization of Block Copolyether

A PPO-PTMG with relatively high *M_n_* (1.20 × 10^4^ Dalton, tested by GPC) and OHV (determined by the titration method, 8.28 mgKOH/g) was selected to be silanized by 3-isocyanatopropyltrimethoxysilane. The silanization of PPO-PTMG is described in detail as follows. In a three-neck flask, 60 g of dried PPO-PTMG was mixed with 0.008 g of bis(lauroyloxy)dioctyltin, 0.004 g of bismuth(III) neodecanoate, and 1.818 g (8.86 mmol) of 3-isocyanatopropyltrimethoxysilane at 100 °C and subjected to constant strong agitation in nitrogen atmosphere for 4 h until the isocyanate group could not be detected in the FTIR spectrum. Then, the silyl-terminated block copolyether (recorded as SiSTP) was obtained.

### 3.5. Measurement

The FTIR spectrum from 400 cm^−1^ to 4000 cm^−1^ was recorded by a VERTEX70 spectrometer (Bruker, Germany). ^1^H-NMR spectra (in CDCl_3_) were obtained on a Bruker AV 400 (400 MHz) instrument (Bruker, Germany). The molecular weight and polydispersity index of the block copolythethers were determined by GPC (Agilent1100 with a differential refractometer as the detector and calculated according to polystyrene standards. The column used in the GPC was Styagel HT provided by Waters Inc.(Shanghai, China), with a range of 500–20,000 Dalton). The analyses were performed at 40 °C, and tetrahydrofuran was used as the eluent at a flow rate of 1.0 mL/min. DSC was conducted using Netzsch DSC 200F3A01 under nitrogen protection at a heating and cooling rate of 5 °C/min and range of −30 °C to 50 °C. 

Determined OHV was obtained by a titration method according to GB/T 12008.3-2009, and the theoretical OHV was calculated as follows:

(1) $$\mathrm{OHV}\;=\;\frac{56.1\times 10^{3}\times 2}{M_{n}}$$

where 56.1, 2, and *M_n_* represent the molecular weight of potassium hydroxide, the theoretical functionality (*f*), and the average molecular weight of copolymer, respectively. Determined *f* is calculated as follows:

(2) $$f\;=\;\frac{\mathrm{OHV}\times M_{n}}{56.1\times 10^{3}}$$

where OHV is the determined value. 

The vulcanizing properties of the modified copolymers including the track free time and the vulcanization rate were separately tested according to GB/T13477.5-2002 and HG/T 4363-2012. The vulcanization system comprised of 10 g of SiSTP or STP-E15, 1.0 g of N-[3-(trimethoxysilyl)propyl]ethylenediamine, 0.3 g of (3-aminopropyl) trimethoxysilane, and 0.1 g of ditin butyl dilaurate.

## 4. Conclusions

(1) Among the dichloromethane, sebacoyl chloride, and m-phthaloyl chloride chain extenders, the highest reactive m-phthaloyl chloride can most effectively couple PPO and PTMG oligomers to form multiblock copolymers with high molecular weight and low crystallinity. 

(2) For the chain extension of PTMG and PPO oligomers using m-phthaloyl chloride, the optimized feed ratio of Et_3_N/COCl/OH was 4/1/1, and the optimized temperature and time were 60 °C and 4 h, respectively. 

(3) A novel silyl-terminated polyether was obtained by the silanization of the block copolymer. The novel silyl-terminated polyether can be used as an alternative and promising base polymer in sealants/adhesives due to its comparable vulcanization properties with commercial silyl-terminated PPO.

In future work, we studied the preparation, properties, and application of sealants based on the novel silyl-terminated polyether developed in this work to verify the superiority of our silyl-terminated polyether as a new type of base polymer in sealants.

## Figures and Tables

**Table 1:** Block copolyethers and their chemical structure information.

Copolymer	Chain Extender	*M_n_*	Đ	OH value (OHV)/mgKOH/g		^c^ *f*	^d^*T_c_* (°C)	^e^*T_m_* (°C)
				^a^ Ther.	^b^ Titr.			
I	CH_2_Cl_2_	7200	1.71	15.52	9.70	1.25	−3.1	25.5
II	sebacoyl chloride	10,700	1.85	10.44	7.54	1.44	−18.3	22.3
III	m-phthaloyl chloride	12,000	1.85	9.29	8.28	1.78	-	21.2

^a^: Theoretical values of OHV; ^b^: Titrimetric values of OHV; ^c^: determined; ^d^: *T_c_* is the maximal crystallization rate temperature; ^e^: *T_m_* is the temperature of crystalline melting.

**Table 2:** Synthesis conditions and structure information of some block copolyethers.

Copolymers	Feed Ratio of Et_3_N/COCl/OH	Temp. (°C)	Time (h)	*M_n_*	Đ	OHV (mgKOH/g)		*f*
						^a^ Ther.	^b^ Titr.	
III	2/1/1	60	4	12,000	1.71	9.29	8.28	1.78
IIIa	4/1/1	60	4	14,800	1.96	7.56	4.16	1.10
IIIb	4/1/1.2	60	4	14,400	1.99	7.74	4.36	1.13
IIIc	4/1/1	50	4	13,600	1.84	8.21	7.08	1.72
IIId	4/1/1	60	2	11,000	1.90	10.15	9.46	1.86

^a^: Theoretical values of OHV; ^b^: Titrimetric values of OHV.

**Table 3:** Experiment designs of different chain extending conditions for optimization.

Reaction Condition Order	Feed Ratios		Temperature (°C)	Time (h)
	Et_3_N/COCl ^a^	COCl/OH ^b^		
1	1/1	0.6/1	50	1
2	2/1	0.8/1	60	2
3	3/1	1/1	70	4
4	4/1	1.2/1	80	6
5	5/1	1.4/1	-	-

^a^ The COCl group was from the m-phthaloyl chloride, and Et_3_N/COCl was the mole ratio of Et_3_N to COCl group; ^b^ OH group was from PPO and PTMG raw materials, and COCl/OH was the mole ratio of COCl to the OH group.
